# Portable Handheld Slit-Lamp Based on a Smartphone Camera for Cataract Screening

**DOI:** 10.1155/2020/1037689

**Published:** 2020-08-01

**Authors:** Shenming Hu, Hong Wu, Xinze Luan, Zhuoshi Wang, Mary Adu, Xiaoting Wang, Chunhong Yan, Bo Li, Kewang Li, Ying Zou, Xiaoya Yu, Xiangdong He, Wei He

**Affiliations:** ^1^College of Medicine and Biological Information Engineering, Northeastern University, Shenyang 110016, China; ^2^He Eye Specislists Hosipital, Shenyang 110000, China; ^3^He University, Shenyang 110000, China; ^4^Shenyang Eyerobo Co., Ltd., Shenyang 110000., China; ^5^Shenyang Eye Industry Technology Research Institute, Shenyang 110000, China

## Abstract

**Purpose:**

As part of plans to provide help to people in remote and poor areas who have no medical resources, a portable slit-lamp based on a smartphone was proposed. This would help in early screening of cataract diseases.

**Methods:**

This means a microlens is designed that would work with a phone's camera. The phone's photo taking function is used in capturing the image of the eyes lens to replace the observation system of the desktop slit-lamp. A simplified slit light band was designed. In order for the light source part to meet the portable requirements of the slit-lamp, the adjustable and diffused light functions of the ligaments were removed in this design. Furthermore, the images collected by the smartphone are uploaded to the deep learning cataract screening system, which can achieve real-time and effective screening of cataract.

**Results:**

Unlike the desktop slit-lamp, which needs skilled personnel to operate, this device can be easily operated by less-skilled or inexperienced doctors. This eliminates the concerns of inaccurate diagnosis based on the use of unskilled professionals. Due to the portability, ease of use, and simplicity in obtaining crystal images of this device, it serves as a promising platform for nonhospital screening and telemedicine.

**Conclusions:**

In this paper, we invented a small portable device for screening cataract. This device is to make screening and diagnosis of cataract in remote areas very fast and effective. It will also solve the problem of inadequate specialized doctors and equipment in those areas as well. *Translational Relevance.* Smartphones can be used with portable slit-lamps to capture the images of the lens.

## 1. Introduction

In recent years, the automatic cataract classification method has seen a lot of development. The continuous upgrading of the algorithm has made the automatic diagnosis of cataract more practical, especially the application of deep learning technology, so that the accuracy of cataract screening is improved [1–4]. With smartphones becoming more popular, their simple and easy to use photo taking functions are being used in ophthalmic screening [5–7]. The function of the smartphone slit-lamp is to provide a portable screening equipment that can easily be used in areas with little or no ophthalmic medical resources without geographical restrictions. With regards to operation, the user only needs about 20 minutes of training because it is simple to operate; therefore, there is no requirement for medical background of the user. The invention of smartphone slit-lamps makes it easier to obtain eye lens images, and it also promotes the progress of deep learning cataract screening technology through big data.

**Figure 1:**
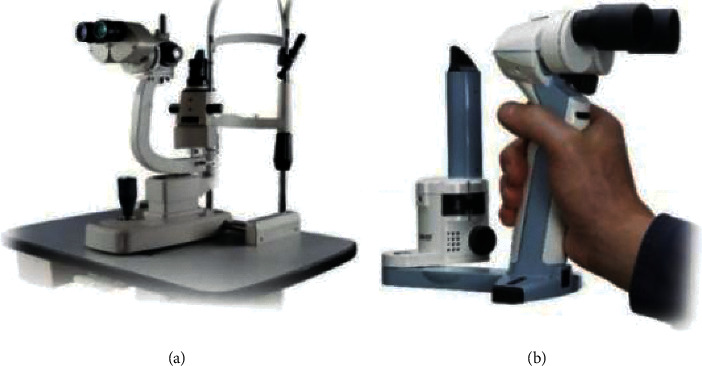
(a) Table-type slit-lamp. (b) Handheld slit-lamp.

**Figure 2:**
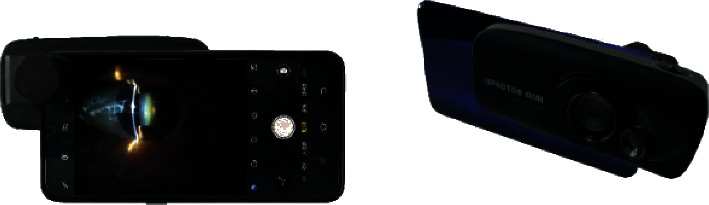
Smartphone slit-lamp installed on a smartphone.

Normally, the eye lens image is obtained using the desktop slit-lamp, as shown in Figure 1(a). The desktop slit-lamp is large and expensive, and it can only be operated by trained technicians in hospitals or clinics. It cannot be used in remote and less-developed areas. The conventional portable slit-lamp is small as shown in Figure 1(b). It is easy to carry but complex, and it cannot be used to capture lens images. A doctor can diagnose cataract disease according to the state of the eye lens irradiated by the portable slit-lamp. For this reason, a small optical device has been invented that can be mounted on a smartphone to facilitate the examination and recording of lens images. A smartphone slit-lamp can effectively screen cataract diseases through an in-depth learning screening system as shown in Figure 2. The era of in-depth learning has made the solution of many tasks simple [8–11]. Therefore, how to reduce the volume of slit-lamp and reduce the difficulty of use while completing the automatic classification of cataracts with real time feedback is the main research focus of this paper. How to design a slit-lamp that meets multidirectional screening is also very important; so this paper will focus on how to design such equipment [12–14]. Figure 1 shows the schematic diagram of desktop slit-lamp and the portable slit-lamp, and Figure 2 shows the schematic diagram of a smartphone slit-lamp installed on a smartphone [15].

## 2. Materials and Methods

### 2.1. Smartphone Slit-Lamp Composition

The smartphone slit-lamp is small in size, portable, and easy to use. Its size is 126 ∗ 53 ∗ 31 mm and weighs about 100 g. This means that it is easy to carry and can be placed in the pocket. The users can be doctors without professional background such as village doctors and grass-root community screening personnel. Before use, they will go through a 20 minutes training on the operation of the smartphone slit-lamp and the software used with the slit-lamp. The trained users can complete the screening themselves. They are not bound by knowledge background, location, or time.

### 2.2. Structure of Smartphone Slit-Lamp

The slit-lamp uses the Tyndall phenomenon to make the narrow band of light pass through the lens of the human eye. We can then observe the light path. This slit light source is called the “light knife.” The slit-lamp in this paper uses the generated light to illuminate the eye lens, and it uses its microlens to increase the illuminated crystal image. This ensures that the images are at a distance of 40 mm. With the smartphone camera function, the images are collected and uploaded to the deep learning system.

**Figure 3:**
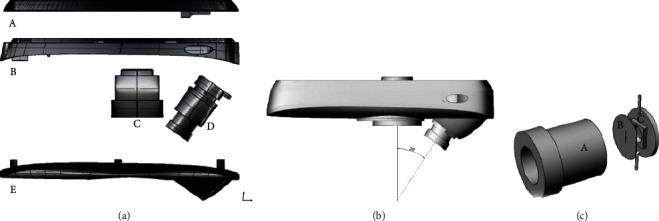
(a) Smartphone slit-lamp components. (b) Smartphone slit-lamp model diagram. (c) Slit light source.

Figure 3 shows the 3D structure of the various components of the smartphone slit-lamp. They include the upper part of the slit-lamp housing (A), the upper and middle parts of the slit-lamp (B), microlens (C), and slit light source (D), which is mainly made up of light source lens A, slit slice B, and LED light source C These parts are simple and easy to assemble. The light from the LED lamp passes through the slit to form a slit light, which is adjusted to about 0.2 mm through the light source lens. It will then scan the lens of the eye and take pictures using the photo taking function of the phone camera. During the shooting, the smartphone lens and the slit-lamp are mounted together using the connection clip. The angle between the slit light source and the microlens is 30 degrees and the imaging focus is 40 mm. By this, a common focus is formed with the microlens. The eye lens is magnified by 10 times, which is similar to the traditional cataract examination method. The light knife is less or equal to 0.2 mm in the 40 mm focus position, and the image of the light knife in common focus with the microhead shows that such a functional design ensures the accuracy of the collected data.

### 2.3. Design Composition of Smartphone Slit-Lamp

A complete slit-lamp is made up of 3 parts: an imaging system, a lighting system, and a bracket. The smartphone slit-lamp also consists of these 3 parts.

#### 2.3.1. Imaging System

**Figure 4:**
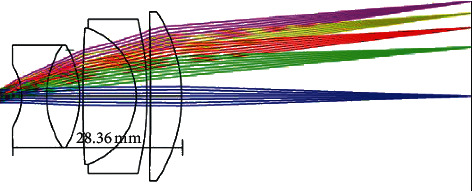
Microlens light path.

The smartphone slit-lamp is designed with a microlens. The main parameters of macrolens design are shown in Table 1:

The light path of the microlens is shown below:

Figure 4 shows the optical path diagram of the microlens. The microlens consists of 3 groups of lenses, 2 double-glued lenses, and 2 convex lenses. The optical path distance is 63.36 mm. The diameter of the aperture of the phone is 1.7 mm, and the lens of the smartphone is placed on the surface of the lens at 3 mm. The direction of light is from right to left, which is a representation of the surface of the human eye when light goes through the lens light path and into the smartphone camera. To accurately obtain the width of 40 mm, approach the human eye with the camera to avoid the fear that the camera is too close. The device design is small in size, simple, and not expensive (for the lens design parameters, refer to the slit-lamp industry standard YY0065-2007, and it has passed the Hangzhou Medical device testing institute slit-lamp inspection standard certification).

**Figure 5:**
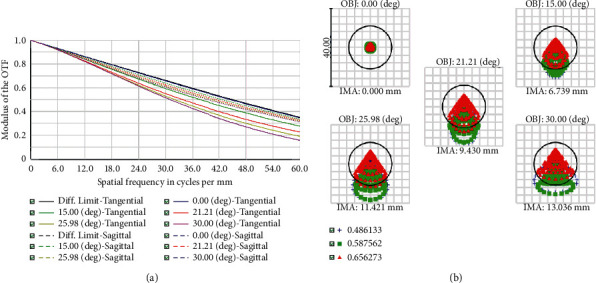
(a) An MTF view of a microlens with an MTF greater than 0.2 over the entire field of view at 60 HZ. (b) Scatter plot showing spot size of lens near diffraction limit.

The microlens ultimately ensures that the slit-lamp can collect high definition images. The MTF of the lens represents the image quality. The scatter plot close to the line emission limit shows high image sharpness as shown in Figure 5.

#### 2.3.2. Lighting System

The source of light is the LED lamp head, which has a slit. An 8 mm focal length lens is used in projecting the light band generated by the light source slit to 40 mm, which is cofocused with the microlens and an angle of 30 degrees to the center of the microlens, as shown in Figure 3(b). This angle is often used in detecting cataract. The width is less than or equal to 0.2 mm.

#### 2.3.3. Bracket

In the structural design, the shell of the smartphone slit-lamp and the microlens are in cofocus. The focus is on the center of the microlens and 40 mm from the human eye to avoid being too close to the object. The reserved distance for the mechanical structure is 3 mm when the lens is connected to the phone.

The smartphone slit-lamp and the traditional desktop slit-lamp are similar. The desktop slit-lamp is used for eye observation, and it has a single light and a smartphone camera design. The desktop slit-lamp light source is able to produce a narrow band and has an adjustable fissure width, diffuse light, and cobalt blue light. As much as possible, the number of movable parts on the original slit-lamp is reduced, so that the operator does not need to set up the equipment before inspection and can operate the slit-lamp and reduce adjustable links thereby increasing the ease of use.

### 2.4. Use of Smartphone Slit-Lamp

**Figure 6:**
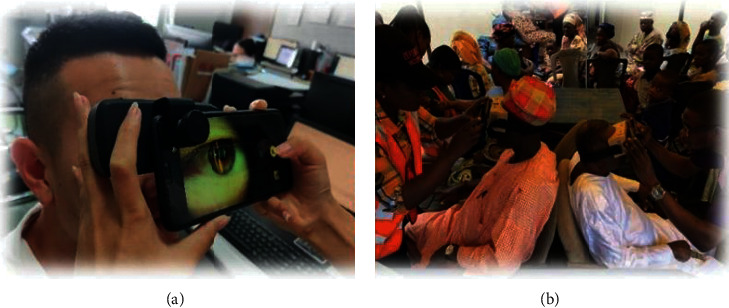
(a) Schematic diagram of slit-lamp use. (b) Screening of cataract diseases in Africa.

To obtain pictures using the camera function of the smartphone, connect the smartphone and scan the light knife of the slit-lamp into the crystal of the human eye. The required lens image can be obtained, and the crystal images of the eye can be seen on the phone. The clarity of the image can affect the diagnosis of the disease, and so, users must ensure that the environment is right. If the environment in which the slit-lamp is being used is too bright, it will affect the quality of the images. In such situation, the patient can wear a dark chamber to avoid image distortion and provide the accurate feedback. The smartphone slit-lamp is suitable for people in underdeveloped areas and places with less medical resources such as some parts of Africa, which are lagging behind in medical care and making it difficult to meet the environmental requirements of the desktop slit-lamp.  Figure 6 shows the use of the smartphone slit-lamp in Nigeria. By using the smartphone slit-lamp, the timely detection and feedback of cataract diseases in poor areas can be accomplished. It has become a very practical ophthalmic examination equipment due to its small size, simplicity, and ease of use.

## 3. Result

**Figure 7:**
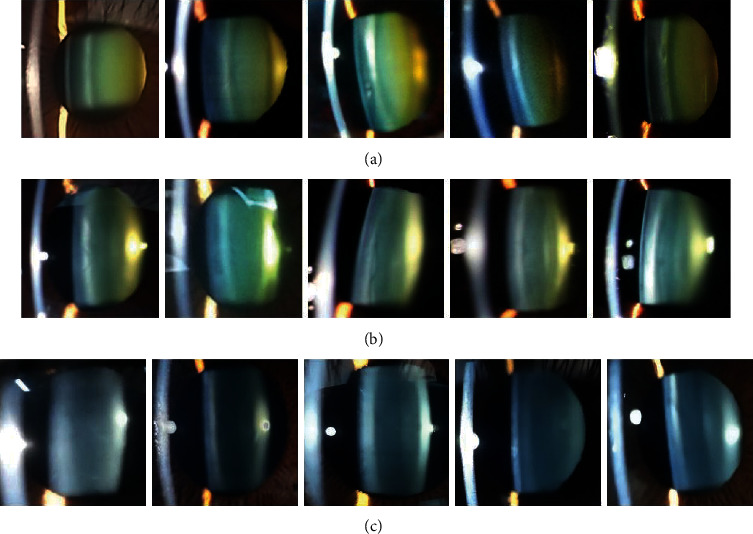
Example diagram of smartphone slit-lamp judging the category of eye crystals.

Figures 7(a)–7(c) show the images of the eye as captured by the smartphone slit-lamp. The images indicate images of cataract patients, lens transparency decline, and normal eyes, respectively. As seen, the images taken by the smartphone slit-lamp are clear. The anterior capsule, cortex, and posterior capsule of the lens of the eye are very easy to differentiate. Lens or cortical opacification is the main basis for cataract diagnosis.

Based on statistics derived from studies, the majority of blindness caused by cataract is higher than that of other causes. It is therefore important that screening and diagnosis of cataract is performed early to avoid blindness caused by cataract, which leads to low quality of life. The lens of the eye can be divided into three according to the degree of opacification: cataract, decrease in lens transparency, and normal. In comparing the three, those with decreased lens transparency are less that the cataract patients and the lens of the normal subjects are transparent and highly transparent. The in-depth learning screening system is responsible for cataract detection and the state of the eye diagram.

The patient is able to make decisions based on the eye chart diagram and feedback result. If it is cataract or decrease in lens transparency, he/she can go to the hospital for further medical treatment according to the situation so as to restore normal health.

## 4. Discussion

The smartphone in recent years has gained much interest in many literatures, especially as tools in ophthalmology. The smartphone slit-lamp serves as an important device for medical diagnosis. It is portable, and it has the capacity to store data and wireless connection function. All those who have gone through training can use it independently, meaning it can be used in economically less-developed areas with ease. It is small and easy to carry. In addition, it obtains clear images, and it is easily compatible with many smartphones due to its connection clip. It is suitable for community cataract screening by nonophthalmic medical personnel who undergo simple training.

In conclusion, the slit-lamp in this paper provides a great potential for its portability, simplicity, and ease of operation, and it also plays an important role in the research and development of artificial intelligence algorithm in real time screening and feedback of cataract.

In economically underdeveloped areas and nonhospital settings, this device proves a more convenient method of screening and the detection of cataract as a result of lack of medical resources, so that people can be treated in time in order to avoid blindness caused by cataract. Furthermore, future advancement such as the use of diffuse light and cobalt blue light will help in observing the cornea, iris, and lens, which will enable the detection of keratitis, corneal ulcer, and damage. For this reason, smartphone slit-lamp is used not only to complete cataract screening but also it can be used in other areas of medicine in the future.

## Figures and Tables

**Table 1:** Main design parameters of microlens.

Focal length	25.98 mm
Total length of the system (total length of the light path of the three sets of lenses)	23.36 mm
Work F/# (imaging capability)	15.28 mm
Height	15 mm
Distance	40 mm
Magnifying power	10

## Data Availability

The data about new portable slit-lamp equipment used to support the findings of this study are available from the corresponding author upon request.

## References

[1] Hall N. F., Lempert P., Zakir R. P., Phillips D. (1999). Grading nuclear cataract: reproducibility and validity of a new method. *British Journal of Ophthalmology*.

[2] Shier L. T., Wolfe J. K., Singer D. M. (1993). The lens opacities classification system III. *Archives of Ophthalmology*.

[3] Li H., Lim J. H., Liu J. An automatic diagnosis system of nuclear cataract using slit-lamp images.

[4] Fan S., Dyer C. R., Hubbard L. (2003). *An Automatic System for Classification of Nuclear Sclerosis from Slit-Lamp Photographs*.

[5] Kaur M., Kaur J., Kaur R. Low cost cataract detection system using smartphone.

[6] Sanguansak T., Morley K., Morley M. (2016). Comparing smartphone camera adapters in imaging postoperative cataract patients. *Journal of Telemedicine & Telecare*.

[7] Sharma A., Subramaniam S. D., Ramachandran K., Lakshmikanthan C., Krishna S., Sundaramoorthy S. K. (2016). Smartphone-based fundus camera device (MII ret cam) and technique with ability to image peripheral retina. *European Journal of Ophthalmology*.

[8] Long E., Lin H., Liu Z. (2017). An artificial intelligence platform for the multihospital collaborative management of congenital cataracts. *Nature Biomedical Engineering*.

[9] Duncan D. D., Shukla O. B., West S. K., Schein O. D. (1997). New objective classification system for nuclear opacification. *Journal of the Optical Society of America A*.

[10] Gao X., Lin S., Wong T. Y. (2015). Automatic feature learning to grade nuclear cataracts based on deep learning. *IEEE Transactions on Biomedical Engineering*.

[11] Huang W., Chan K. L., Li H. (2010). A computer-assisted method for nuclear cataract grading from slit-lamp images using ranking. *IEEE Transactions on Medical Imaging*.

[12] Rif’ati L., Basuki B. (2013). Slit-lamp calibration, crucial but neglected. *Health Science Journal of Indonesia*.

[13] Graether J. M., Marshalltown I. A (2011). *Slit-lamp Photo Assembly*.

[14] Yamamoto S., Manabe K. N., Yamamoto K. (2010). High-definition slit-lamp video camera system. *Ophthalmic Surgery, Lasers, and Imaging*.

[15] Download Link of Smartphone Application Program, http://47.104.91.122/download/ruimu.apk

